# Emerging evidence for beneficial macrophage functions in atherosclerosis and obesity-induced insulin resistance

**DOI:** 10.1007/s00109-016-1385-4

**Published:** 2016-02-04

**Authors:** Timothy P. Fitzgibbons, Michael P. Czech

**Affiliations:** Cardiovascular Division, Department of Medicine, University of Massachusetts Medical School, 55 Lake Avenue North, Worcester, MA 01655 USA; Program in Molecular Medicine, University of Massachusetts Medical School, Worcester, MA 01655 USA

**Keywords:** Visceral adipose, Atherosclerosis, Insulin resistance, Autophagy, Macrophage, Cholesterol, mTOR

## Abstract

The discovery that obesity promotes macrophage accumulation in visceral fat led to the emergence of a new field of inquiry termed “immunometabolism”. This broad field of study was founded on the premise that inflammation and the corresponding increase in macrophage number and activity was a pathologic feature of metabolic diseases. There is abundant data in both animal and human studies that supports this assertation. Established adverse effects of inflammation in visceral fat include decreased glucose and fatty acid uptake, inhibition of insulin signaling, and ectopic triglyceride accumulation. Likewise, in the atherosclerotic plaque, macrophage accumulation and activation results in plaque expansion and destabilization. Despite these facts, there is an accumulating body of evidence that macrophages also have *beneficial* functions in both atherosclerosis and visceral obesity. Potentially beneficial functions that are common to these different contexts include the regulation of efferocytosis, lipid buffering, and anti-inflammatory effects. Autophagy, the process by which cytoplasmic contents are delivered to the lysosome for degradation, is integral to many of these protective biologic functions. The macrophage utilizes autophagy as a molecular tool to maintain tissue integrity and homeostasis at baseline (e.g., bone growth) and in the face of ongoing metabolic insults (e.g., fasting, hypercholesterolemia, obesity). Herein, we highlight recent evidence demonstrating that abrogation of certain macrophage functions, in particular autophagy, exacerbates both atherosclerosis and obesity-induced insulin resistance. Insulin signaling through mammalian target of rapamycin (mTOR) is a crucial regulatory node that links nutrient availability to macrophage autophagic flux. A more precise understanding of the metabolic substrates and triggers for macrophage autophagy may allow therapeutic manipulation of this pathway. These observations underscore the complexity of the field “immunometabolism”, validate its importance, and raise many fascinating and important questions for future study.

## Background

The idea that inflammation, and specifically the macrophage foam cell, is vital to the initiation and progression of atherosclerosis dates back to initial observations by the pathologist Rudolph Virchow and studies in rabbits in 1958 [1]. A seminal paper by Hotamisligil and colleagues in 1993 demonstrated increased tumor necrosis factor alpha (TNF-α) messenger RNA (mRNA) expression in the visceral adipose of obese mice and neutralization of TNF-α led to improved insulin stimulated glucose uptake [2]. They postulated that there may be a link between obesity, insulin resistance (IR), and inflammation [2]. However, the cellular source of TNF-α was not clear until 2003, when two simultaneously published papers demonstrated that obesity results in inflammation and macrophage accumulation in epididymal adipose tissue [3, 4]. Since that time, there have been hundreds of papers demonstrating that abrogation of inflammation can improve or prevent atherosclerosis or obesity-associated insulin resistance (IR) in mice. Searching PUBMED for the terms “myeloid specific knockout AND metabolism” returns 2259 references, half of which have been published in the last 5 years. There are many excellent reviews on the adverse effects of chronic inflammation in these diseases and that will not be the focus of this paper [1, 5, 6]. However, the fact that clinical trials of anti-inflammatory medications in diabetes have not been overwhelmingly positive suggests that the pathophysiology is more complex than it may appear [7].

In normal conditions, both arteries and adipose tissue have a population of resident macrophages [8]. White adipose tissue (WAT) includes the subcutaneous or inguinal (iWAT) and visceral adipose tissue (VAT), the latter being more prone to inflammation. VAT includes the epididymal (eWAT), parametrial (pWAT), and mesenteric (mWAT) depots, among others. Macrophages exert multiple and varied effects in these tissues depending upon the physiologic conditions. Their number and activity is in constant flux, via recruitment of monocytes from the blood, proliferation of local tissue macrophages, egress to lymphatics, and cell death. This makes studies examining an endpoint at a single point in time (e.g., atherosclerosis or IR after 12 weeks of high fat diet (HFD)) very difficult to interpret. For example, silencing of TNFα expression in the eWAT macrophages of ob/ob mice improves glucose tolerance [9]. Yet, silencing of lipoprotein lipase (LPL) in eWAT macrophages reduces foam cell formation and worsens glucose tolerance [10]. Furthermore, although short-term HFD (less than 1 week) results in obesity and eWAT inflammation, IR is not improved by depleting macrophages or lymphocytes under these conditions [11]. In fact, it appears that the adipocyte plays a cell-autonomous role in the development of IR that is dependent primarily upon cell size but also its metabolic substrates (saturated vs. unsaturated fatty acids) [12]. Hence, the role that macrophages play in IR and other pathologies is very complex, dependent upon time, context, and other host-specific factors.

### Defining macrophages and activation states

**Fig. 1:**
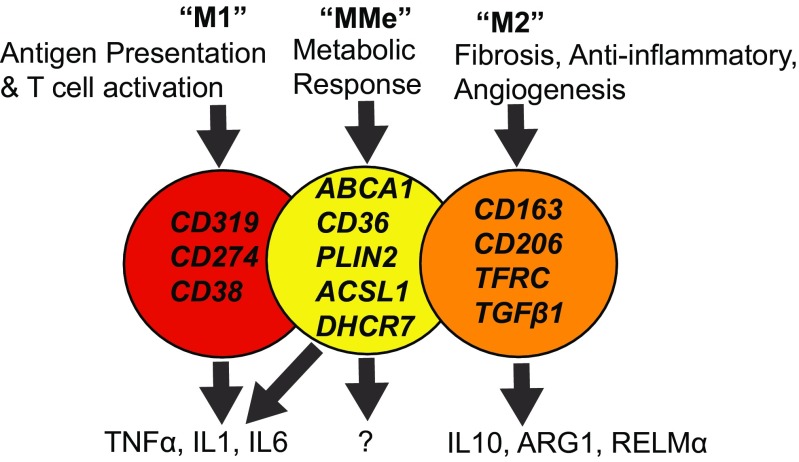
The spectrum of macrophage polarization. Although macrophage function has typically been dichotomized into classical (M1) or alternative (M2), recent data highlights the limitations of this classification and underscores the potential functional utility of “metabolically” (MMe) activated subsets [13, 14, 30]. Such a subset is characterized by proteins responsible for lipid uptake (CD36), storage (PLIN2), and export to high-density lipoproteins (ABCA1)

Traditionally, macrophage polarization has been dichotomized into classical (M1) or alternative (M2) activation. These distinctions were developed based upon the in vitro response of macrophages to INFγ or IL4 and IL13, respectively [13]. We now know that this is an oversimplification, and that in vivo macrophages are very adaptable and exhibit a broad range of activation states*.* Nonetheless, this is a useful framework that we shall expand upon. M2 activation is a characteristic feature of tissue resident macrophages; which account for approximately 10–15 % of stromal cells in lean eWAT [13]. M2 macrophages remodel extracellular matrix (ECM), regulate angiogenesis, and have anti-inflammatory effects, essential functions for normal growth of adipose tissue (Fig. 1). They secrete anti-inflammatory cytokines such as IL10 and are dependent upon the transcription factors STAT6, IRF-4, and PPARγ for their development. In mice, tissue resident M2 macrophages are generally characterized by the cell surface markers F480^+^, CD11b^+^, and CD11c^-^ [14]. In obesity, blood monocytes infiltrate eWAT, at least partially via CCR2 receptors in response CCL2 ligand, and differentiate into M1 macrophages (F480^+^, Cd11b^+^, and Cd11c^+^ cells) [14]. Again, it should be emphasized that these distinctions are very complex, dynamic, and time dependent. For example, there are at least three populations of recruited interstitial macrophages after 8 weeks of HFD (Mgl1^+^/CD11c^-^, Mgl1^Med^/CD11c^+^, and Mgl1^-^/CD11c^+^) [15]. The latter subset is typically M1 polarized and associated with “crown-like structures”; but many CD11c + cells express both M1 and M2 type mRNAs, and M2 polarization increases with longer duration of HFD (12 weeks) [15]. It is still helpful to think of M1 macrophages as those responding to a Th1 response, with anti-microbial activity, and expressing inflammatory cytokines such as TNFα, IL1β, and IL6 (Fig. 1). M1 activation of macrophages is dependent upon the transcription factors STAT1 and IRF-5. Deletion of CCR2 on circulating monocytes reduces eWAT macrophage accumulation in obese mice by 40 % [16]. Therefore, although this chemotactic pathway is important, there must be additional adipose-specific cues that stimulate inflammation [16]. The fact that many CD11c^+^ cells surround dying adipocytes (epididymal and mesenteric) in “crown-like structures” suggests that the necro-apoptotic adipocyte may be one such cue [17, 18]. In addition to infiltration, proliferation of tissue resident macrophages also contributes to a local pro-inflammatory subset in eWAT and atherosclerotic plaques [19, 20]. The net result is a dramatic increase in the total number of macrophages in obese as compared to lean eWAT, numbering 45–60 % of the stromal fraction [13]. Chronic inflammation in eWAT results in physiological dysfunction of adipocytes, excess free fatty acid release, hepatic and systemic inflammation, and ectopic lipid deposition (liver, heart, skeletal muscle) [21]. Many studies have contributed to this notion; for example, ablation of CD11c + macrophages improves HFD-induced IR [22].

### M2 polarization and adaptive thermogenesis

The importance of tissue resident M2 macrophages in adipose tissue biology has been highlighted by a growing body of literature that demonstrates their role in supporting brown adipose tissue (BAT) function [23–27]. In contrast to WAT, which stores triglyceride, BAT oxidizes fatty acids via uncoupled oxidative phosphorylation to generate heat via non-shivering thermogenesis. A third type of adipocyte, termed the “beige” or “brite” adipocyte, is found in WAT depots, but can be recruited via catecholamines to induce a thermogenic gene program (e.g. UCP1, PGC1α). The activation of brown or “beige” adipocytes hold great promise as a treatment for obesity and there is growing interest in finding methods to stimulate their growth/function. Interestingly, exposure to the cold results in M2 activation of tissue resident macrophages in iWAT and BAT [25]. In fact, these resident cells may be required for expression of a thermogenic gene program in BAT and lipolysis of triglyceride in iWAT, which is the fuel for BAT thermogenesis [25]. Whereas it has always been assumed that sympathetic innervation of fat depots provided catecholamines to stimulate lipolysis and thermogenesis, M2 ATMs induce tyrosine hydrolase (TH) upon cold exposure and may produce, surprisingly, a significant amount of adipose tissue catecholamines [25]. Depletion of BAT tissue resident macrophages via clodronate, or abrogation of M2 polarization in IL4 receptor knockout macrophages (*IL4Rα*^*LoxP/LoxP*^*LysM*^*Cre*^ mice), reduced induction of thermogenic genes in BAT and prevented maintenance of core body temperature upon exposure to 4 °C [25]. More recent work has unraveled a network of immune cells that may orchestrate this process [26, 27]. M2 polarization of ATMs is promoted by paracrine secretion of IL4 by local eosinophils. Eosinophils, in turn, may be stimulated by endocrine circulating factors released from muscle or fat, during exercise or cold exposure, respectively [26, 27]. Reducing environmental temperature from 28 to 4 ° C dramatically alters the BAT transcriptome [28]. Among the many regulated gene networks, upregulation of AMPK and downregulation of mTOR signaling pathways was noted [28]. Thus, cold stress is analogous to fasting conditions and could potentially stimulate autophagic pathways in macrophages or other cells (see below). To summarize, just as prevention of M1 macrophage polarization or infiltration may be beneficial to adipose tissue metabolism, stimulation of M2 polarization in ATMs may be beneficial in iWAT and BAT to promote adaptive thermogenesis and energy expenditure.

### The role of adipocyte autonomous inflammation

Whereas decreasing monocyte recruitment to VAT is beneficial in mouse models of DIO, Asterholm et al. recently published a provocative paper which demonstrates that reducing inflammatory chemokines elaborated by adipocytes themselves during short-term HFD impairs adipose tissue growth and expansion, and results in a worsening of IR [29]. The authors used three different mouse models to abrogate inflammation specifically in adipose tissue. First, HFD-fed mice expressing a dominant negative TNFα (dnTNFα) transgene driven by the aP2 promoter were leaner but more IR than control littermates [29]. This was thought to be due to decreased adipogenesis in the eWAT and iWAT of dnTNFα mice. In a second model, the authors expressed a novel adipose-specific transgene (RID) that broadly inhibits canonical pro-inflammatory pathways. RID mice were also lean and had smaller iWAT, eWAT, and mWAT depots. Interestingly, there was greater fibrosis in the RID adipose tissue, suggesting that a function of acute low-grade inflammation in adipose tissue is to allow for degradation of extracellular matrix (ECM) and adipogenesis [29]. Finally, they utilized inducible expression of IκBα to inhibit NFκB signaling specifically in adipocytes. In comparison to wild type mice, transgenic mice had decreased eWAT weight, increased liver weight, and were more glucose intolerant after 8 weeks of HFD [29]. The authors concluded that adipocyte initiated inflammation is necessary for adipogenesis, because macrophages are needed to remodel ECM and promote angiogenesis, allowing for fat pad growth [29]. There may be depot-specific differences in the role of adipocyte inflammation; for example, inflammation in mWAT stimulates angiogenesis and growth of this depot, allowing for proper intestinal barrier function in obese conditions [29].

### “Metabolic” activation of macrophages

In addition to this beneficial role of inflammation in adipose tissue biology, recent evidence suggests that our definition of “inflammation” in metabolic contexts may be incomplete [30]. Using a proteomics approach to study M1 macrophage cell surface proteins in vitro, Kratz et al. found a panel of three markers for M1 activation that are common to human and mouse macrophages; CD274, CD38, and CD319 [30] (Fig. 1). These three markers were validated on macrophages from the airway of patients with cystic fibrosis, which are chronically exposed to bacterial pathogens. However, none of these markers were increased in the stromal fraction of VAT from obese humans (omental) or mice (eWAT), despite increased mRNA levels of TNFα and IL1β from these same samples [30]. The authors hypothesized that obesity does not induce M1 macrophage polarity per se, but perhaps a distinct macrophage phenotype [30]. By exposing macrophages to high levels of palmitate, insulin, and glucose, they found a panel of markers (*Abca1, Cd36, Plin2*) that were unique for “metabolic activation (MMe)”, and did not overlap with known M1 or M2 markers. Other groups have also found that markers of pro-inflammatory macrophages (M1) are not as abundant in obese VAT as originally thought, especially when total macrophage number is accounted for [3, 4, 14]. Expression of the consensus MMe markers was validated in VAT from obese humans and mice, and their induction was shown to be independent of type 1 interferon or toll-like receptor signaling [30]. Instead, PPARγ and sequestosome-1 (p62) regulated expression of the MMe markers CD36, ABCA1, and PLIN2 [30]. p62^-/-^ macrophages, and macrophages treated with the PPARγ antagonist T0070907, failed to induce MMe markers after palmitate treatment and overproduced TNFα and IL1β. Therefore, there may be a link between metabolic activation and anti-inflammatory effects. The role of PPARγ was not surprising, given its known effects as a regulator of metabolic and anti-inflammatory pathways. However, the mechanism by which p62 suppresses expression of TNFα and IL1β was less clear. p62 is normally degraded in the autophagolysosome and accumulates when autophagy is blocked [30]. Upregulation of p62 suggested that autophagy was activated but flux was reduced in MMe macrophages; this upregulation was correlated with palmitate uptake. Indeed, increasing palmitate concentrations increased the levels of p62, in a CD36-independent manner [30]. To summarize, there appears to be an additional subset of macrophages activated by metabolic factors (MMe) that is distinct from traditional M1 or M2 classifications (Fig. 1). The accumulation of palmitate in macrophage autophagolysosomes may contribute to the anti-inflammatory properties of this novel subset. The precise mechanism of palmitate uptake, and the biological significance of MMe ATMs, has yet to be elucidated.

### Macrophage autophagy and lipid metabolism

There are three main types of autophagy [31–33]. Macroautophagy refers to the engulfment of cytosolic contents into double membrane vesicles called autophagosomes. This process can be selective or non-selective [31]. A second type of autophagy is microautophagy, which is the direct phagocytosis of cytosolic material by the lysosomal membrane. Finally, a third form of autophagy is termed chaperone-mediated autophagy (CMA). Specific motif-containing proteins are recognized by chaperones such as heat shock cognate protein of 70 kDa (hsc70) and then delivered to the surface of the lysosome [31]. CMA has recently been shown to contribute to lipid droplet metabolism, although the degradation of lipids by selective macroautophagy, or “lipophagy”, is thought to be most operative in metabolic disease, and that is the process that we refer to here [34–36]. The findings by Kratz et al. and others suggest that the macrophage utilizes autophagy to metabolize lipid in conditions of fatty acid excess [14, 30, 37]. How and why might this occur?

**Fig. 2:**
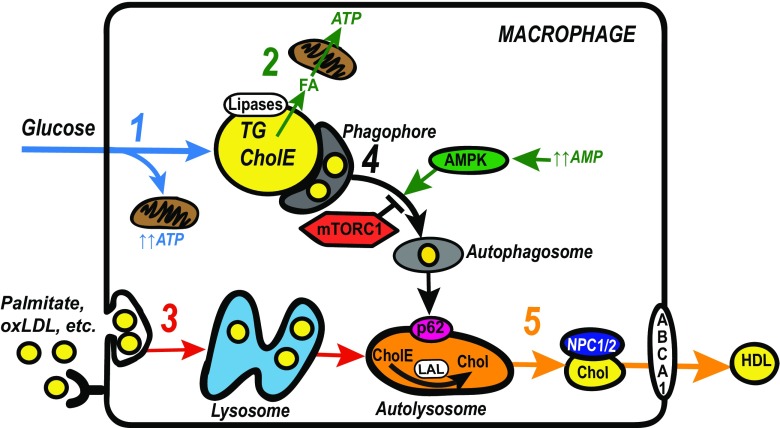
Regulation of lipid metabolism in foam cells and tissue resident macrophages. (step 1, *blue*) In basal conditions, macrophages take up glucose to generate energy via oxidative metabolism, storing excess glucose in lipid droplets after de novo lipogenesis. (step 2, *green*) In fasting conditions, neutral lipases at the lipid droplet surface hydrolyze stored triglyceride (TG) to fatty acids (FA), which undergo beta-oxidation in adjacent mitochondria to generate adenosine triphosphate (ATP). (Step 3, *red*) In obesity or hypercholesterolemia, macrophages in the plaque or visceral adipose tissue may endocytose lipoproteins/lipid via scavenger receptors (CD36, SRA) or via micro pinocytosis. Engulfed lipoproteins are metabolized via lysosomal degradation. (step 4, *black*) Some stored lipid may also be metabolized via autophagy, which is initiated by formation of a phagophore. AMP activated protein kinase (AMPK) and mammalian target of rapamycin (mTORC1), respectively, activate and inhibit this process. The autophagosome fuses with the lysosome to generate the autolysosome. (step 5, *orange*) In the autolysosome lysosomal acid lipase (LAL) hydrolyzes cholesterol ester (CholE) to cholesterol (Chol). Whether or FA generated from the hydrolysis of CholE or TG are available for beta-oxidation is not yet known (not shown). However, the export of cholesterol is dependent on this process. The export of cholesterol is facilitated by the proteins of Niemann-Pick disease, type C1,2 (NPC1/2), and member 1 of human transporter subfamily ABCA (ABCA1). Depending upon the substrates within the autolysosome autophagy may be blocked at this step. This will result in the accumulation of sequestosome-1 (p62) and lipid in the autolysosome and prevent cholesterol efflux to high density lipoprotein (HDL). Decreased autophagic flux will also cause leakage of autolysosome contents into the cytoplasm (cathespins, reactive oxygen species) and cause activation of the inflammasome or unfolded protein response. These latter effects contribute to worsening of local inflammation or programmed cell death

In fasting conditions, when adipocyte lipolysis is maximally activated, there is a sudden and abundant increase in eWAT lipid-laden ATMs [38, 39] (Fig. 2). It is thought that these macrophages are recruited to buffer the acute local increase in FFA concentrations because depletion of macrophages after a 24 h fast results in a dramatic rise in plasma FFA [38]. Whether or not macrophage autophagy is activated in these circumstances is not known.

On the other hand, autophagy may be stimulated in eWAT ATMs during obesity. This possibility was recently raised by Xu et al., who performed microarrays of eWAT in lean and obese mice and correlated the results with three phenotypes: body mass, fasting insulin, or macrophage-specific gene expression [14]. Different transcriptional networks were regulated for each condition, but only one was upregulated in all three; lysosomal biogenesis [14]. Proteins, important for autophagy and lysosomal biogenesis, were enriched in macrophages from the stromal fraction of eWAT from obese mice. In comparison to lean mice, ATMs from obese mice doubled their lysosome content, as measured by Lysotracker accumulation (26 vs. 57 %). Although lysosomes accumulated in both F480^+^/Cd11b^+^ and F480^+^/Cd11b^+^/Cd11c^+^ cells, they were greater in F480^+^/Cd11b^+^/Cd11c^+^ macrophages. Microscopic analysis of these lipid-laden cells demonstrated two classes of lipid droplets: larger bodipy positive droplets with and without surrounding lysosomes and smaller more numerous droplets that co-localized with lysosomal markers [14]. Exposure of bone marrow-derived macrophages to eWAT in vitro induced expression of genes involved in lipid uptake (*Msr1, Plin2*) and lysosomal biogenesis (*Atp6v0d2, Lipa, Ctsk*), but not classic M1 activation (*TNFα, Tlr2, Tlr4*) [14]. Inhibition of lysosomal function using either chloroquine or Bafilomycin A1 resulted in a marked accumulation of macrophage lipid content. These results were consistent with the subsequent studies by Kratz et al., suggesting that obesity results in a unique activation profile of ATMs, characterized by induction of lipid uptake and lysosomal pathway encoding genes [30]*.*

These data suggest that macrophages in eWAT take up and store lipid released from dysfunctional adipocytes, leading to the formation of macrophage “foam cells”. Stored lipid is likely metabolized predominantly by lysosomal degradation; however, it is possible that stored lipid is also metabolized by selective macro-autophagy or “lipophagy”. This process was originally described by Singh et al. in 2009 [40]. Hepatocytes deficient in autophagy-related protein 5 (*Atg5)*, when challenged with oleic acid, have markedly enlarged lipid droplets. Animals with hepatocyte-specific autophagy-related protein 7 (*Atg7*) deletion developed marked hepatosteatosis due to blocked autophagy and resultant reduced lipolysis [40]. Therefore, autophagy appears to serve a protective effect in the liver by preventing hepatosteatosis in the setting of HFD. Furthermore, this protection is at least partially mediated by lysosomal acid lipase (LAL) and not only neutral cytoplasmic lipases (HSL, Nceh1, CEH) [41, 42]. Autophagy in preadipocytes regulates adipogenesis, and its inhibition prevents adipocyte differentiation in vitro and in vivo [43]*.* Whole body *Atg5*^*-/-*^ and *Atg7*^*-/-*^ mice die at birth, but adipocyte-specific ablation of *Atg7*^*-/-*^ results is reduced in leaner, more insulin-sensitive mice, with more brown adipose tissue [43, 44]*.* Whether or not macrophage specific loss of autophagy exacerbates IR in obese mouse models is not yet known. However, there is abundant recent evidence demonstrating that macrophage autophagy is protective against atherosclerosis [37, 41, 45].

### Macrophage autophagy and atherosclerosis

The idea that macrophage autophagy might be beneficial in limiting atherosclerosis stemmed from the observation of autophagosomes in atherosclerotic plaque on electron microscopy [46]. Additionally, as defective efferocytosis and the accumulation of cholesterol crystals are hallmarks of the atherosclerotic plaque, it is intuitive that autophagy may be protective, by potentially clearing this cellular debris. Finally, interventions that activate autophagy, such as fasting and treatment with the mTOR inhibitors everolimus and sirolimus, reduce atherosclerosis in mice [37]*.*

Razani et al. tested the hypothesis that autophagy defective mice on the apoliprotein E null (*Apoe*^*-/-*^) background might develop worse atherosclerosis [37]. *Beclin-1/Atg6* heterozygous deficient mice, which are only haploinsufficient for autophagy, did not develop worse atherosclerosis than *Apoe*^*-/-*^ littermates. However, macrophage-specific *Atg5* knockout mice on the *Apoe*^*-/-*^ background (*Atg5-mϕ Apoe*^*-/-*^) developed dramatically worse atherosclerosis. Reduced autophagic flux was demonstrated by increased p62 protein levels in the aortas of *Atg5-mϕ Apoe*^*-/-*^ mice. In addition to the increased atherosclerotic area, *Atg5-mϕ Apoe*^*-/-*^ mice hyper-secreted IL1β in response to LPS and had markedly reduced cholesterol efflux [37].

It is notable that cholesterol crystals in advanced plaques not only activate the inflammasome but also block autophagic processing. This is reminiscent of the effect of palmitate to inhibit autophagy and subsequently repress the anti-inflammatory effects of autophagy [30]. Hence it appears that the metabolic substrates of autophagy, depending upon their suitability for lysosomal degradation, may determine autophagic flux. Poorly digestible substrates (oxidized LDL, lipofuscin, ceroid) will result in stalled autophagy and stimulation of pro-inflammatory pathways [30, 37, 47]. For example, the accumulation of undigested cholesterol crystals stimulates the inflammasome, whereas palmitate and other saturated fatty acids activate the unfolded protein response [34, 48]. In this way, atherosclerosis, obesity, and hepatosteatosis may all represent acquired lysosomal storage disorders [49]. Compounds that promote the mobilization of cholesterol crystals from the lysosome and into the cytoplasm (e.g.,**β**-cyclodextrins) have been promoted as potential therapeutic compounds for these disorders [50–52]. This is in contrast to oleate and other unsaturated fatty acids that stimulate, but do not stall, flow through the autophagy pathway [34].

Stalled autophagy not only triggers inflammation and lipid droplet accumulation, but it prevents the hydrolysis of cholesterol ester and subsequent efflux of cholesterol to apo-A1 [41]. It was previously thought that cytoplasmic neutral cholesterol esterases (HSL, ATGL) were responsible for hydrolysis and mobilization of cholesterol from foam cells. However, Ouitmet et al. showed that in lipid-loaded macrophages, cholesterol efflux to apoA-1 is dependent on lysosomal acid lipase (LAL) [41]. An inhibitor of LAL (lalistat) prevented cholesterol efflux to apo-A1. RCT in vivo was reduced in mice given lipid-loaded macrophages from *Atg5*^*-/-*^ donors. Therefore, mobilization of cholesterol for RCT from foam cells is dependent upon autophagy. Foam cells accumulate lipid droplets in an unregulated fashion via non-selective uptake of triglyceride rich lipoprotein particles (TRLP) by scavenger receptors (SR-A1, SR-B1, CD36), whereas non-lipid laden macrophages take up TRLP in a regulated fashion, via lipoprotein receptors (LDLR). How do lipid-laden macrophages sense the metabolic milieu and regulate autophagic pathways?

### MTOR links environmental cues to autophagy and nutritional status

Mammalian target of rapamycin (mTOR1) provides a link between the presence of extracellular nutrients/growth factors and the stimulation of cell growth and protein synthesis [53]. Stimulation of the insulin receptor leads to activation of phosphatidylinositiol-3 kinase (PI3K), phosphorylation of Akt, and downstream activation of mTOR1. It is important to note that the effect of PI3K on autophagy is class dependent; class 1 PI3 kinases inhibit autophagy (by activating mTOR1), whereas class 3 PI3 kinases activate autophagy by inducing beclin 1 [54]. Activation of mTOR1 inhibits autophagy by several mechanisms. mTOR1 phosphorylates ATG13 and ULK1/2, which are components of the autophagy initiating UNC-5 like autophagy activating (ULK) complex [55]. mTOR1 also phosphorylates ATG14L, causing inhibition of the associated class 3 PI3 kinase VPS34. Finally, mTOR1 phosphorylates autophagy/beclin 1 regulator 1 (AMBRA1) [46, 55, 56]. In this way, mTOR activation signals cells that environmental conditions favor cellular growth, and autophagy or “self-digestion” is not necessary for the provision of metabolites for cellular growth and ATP synthesis [46]. In contrast, under fasting conditions, when there are lower amounts of extracellular nutrients and growth factors, mTOR1 is not activated, and *Atg1* kinase activity is derepressed, therefore stimulating autophagy for the provision of cellular nutrients. The importance of this pathway to cellular metabolism is reflected in its inter-species conservation, most dramatically seen in *C.elegans*, where fasting and stimulation of autophagy promote longevity and delay cellular senescence [57]. These insights have led to the understanding that reduced insulin signaling promotes longevity and might have other beneficial effects*.*

Interestingly, deletion of the insulin receptor specifically from macrophages, as opposed to whole body deletion, improves atherosclerosis on the *Apoe*^*-/-*^ background [58]. In contrast, hepatic insulin receptor deletion promotes atherosclerosis even in wild type mice, due to the development of “selective” insulin resistance [59]. Hepatic insulin receptor knockout stimulates VLDL synthesis resulting in high blood triglyceride-rich lipoprotein levels and atherosclerosis. deletion of the insulin receptor in macrophages results in decreased atherosclerosis and inflammatory cytokine production by macrophages, despite identical blood lipoprotein levels and metabolic features [58].

Myeloid-specific insulin receptor knockout also provides protection against DIO [60]. The mechanism was thought to be due to increased macrophage apoptosis in the absence of insulin and/or decreased macrophage infiltration into adipose tissue [60]. We speculate that an additional protective effect of myeloid specific insulin receptor knockout may be due to reduced mTOR1 activation and resultant stimulation of autophagy*.* Numerous other studies in mice and rabbits have demonstrated that inhibition of mTOR1 signaling by pharmacologic (rapamycin analogs) or genetic approaches (mTOR1 siRNA) leads to stimulation of autophagy, a reduction in atherosclerotic lesion size, and stabilization of the fibrous plaque [46, 54].

The protective functions of macrophage autophagy extend beyond “lipophagy” in the atherosclerotic plaque or VAT. Autophagy also helps to prevent apoptosis, promote efferocytosis of necrotic cells, and digest cellular components (e.g., mitochondria) damaged by oxidative stress [61]. For example, 7-ketocholesterol has been shown to induce autophagy and prevent induction of smooth muscle cell apoptosis by fluvastatin, through yet to be identified mechanisms [62]. The cardioprotective effects of adiponectin (Acrp30) may be partially due to the stimulation of autophagy in foam cells of the atherosclerotic plaque [63]. Transplant of Acrp30-deficient perivascular fat to the carotid artery of *Apoe*^*-/-*^ mice results in worse atherosclerosis and decreased macrophage autophagy; Acrp30 also stimulates autophagy in macrophages in vitro [63]. This effect of Acrp30 may be mediated by AMP-activated protein kinase (AMPK), a known antagonist of mTOR1 signaling whose activity is increased by fasting [53]. AMPK mediates these effects by activating tuberous sclerosis complex 2 (TSC2) and also by direct serine phosphorylation of raptor [53].

## Conclusions

For over a decade, there has been fervent research aimed at deciphering the effects of inflammation on the pathophysiology of atherosclerosis and obesity-associated IR. The lipid-laden macrophage is the central orchestrator of the innate immune response to modified lipoproteins in the atherosclerotic plaque and FFAs in obese VAT depots. There is little doubt that chronic inflammation incited by the lipid-laden macrophage contributes significantly to the pathophysiology of these disorders [22, 64, 65]. However, there is increasing evidence that macrophages also play beneficial roles in the prevention of atherosclerosis and obesity-induced IR. These beneficial effects relate to the maintenance of tissue homeostasis, many of which are mediated by macrophage autophagy. Examples of such effects include the buffering and metabolism of excess local lipids, efferocytosis of necrotic cells and debris, and the prevention of apoptosis. Macrophages in both atherosclerosis and diet-induced obesity have features of acquired lysosomal storage disorders (e.g., p62 accumulation), that may be precipitated by specific metabolic substrates (e.g., oxLDL, palmitate). Furthermore, the beneficial effects of macrophage autophagy are highlighted by worsening of atherosclerosis in macrophage-specific knockouts deficient in this pathway. Such models have yet to be described in obesity. Nonetheless, we believe that there is sufficient data to warrant investigation of pharmacologic (mTOR1 inhibitors, AMPK activators, cyclodextrins) or dietary interventions (fasting) that may stimulate macrophage autophagy and possibly represent a novel pathway to prevent or treat cardiometabolic disorders.
